# Molecular Factors Involved in the Pathogenesis of Pyometra in Domestic Cats (*Felis catus*)

**DOI:** 10.3390/ani14202987

**Published:** 2024-10-16

**Authors:** Acácia Eduarda de Jesus Nascimento, Luciano Cardoso Santos, Juneo Freitas Silva

**Affiliations:** Centro de Microscopia Eletronica, Departamento de Ciencias Biologicas, Universidade Estadual de Santa Cruz, Campus Soane Nazare de Andrade, Ilheus 45662-900, Brazil; acaciaeduarda1@gmail.com (A.E.d.J.N.); lucianouesc280@gmail.com (L.C.S.)

**Keywords:** feline pyometra, hormonal disorders, uterine infection, molecular factors

## Abstract

Pyometra is a major reproductive disorder affecting female cats. Characterized by the accumulation of purulent exudate in the uterus, it can lead to severe and potentially fatal complications. This pathology primarily occurs in adult cats during diestrus, but can also affect younger felines, often due to inappropriate administration of hormonal medications. While the etiopathogenesis of pyometra is better understood in bitches, few studies have addressed the molecular factors involved in feline pyometra. Therefore, this review aims to provide an up-to-date and comprehensive discussion of pyometra in cats, focusing on the regulation of molecular factors described in this species, including hormonal, redox, immunological, and growth factors. Understanding the regulation of these factors in pyometra is crucial for elucidating its pathogenesis in domestic cats and for improving diagnosis and identifying novel therapeutic approaches.

## Simple Summary

This article reviews the molecular factors causing pyometra in domestic cats, a serious uterine infection. While well-studied in dogs, pyometra in cats is less understood. The focus is on hormonal, redox, immunological, and growth factors, aiming to improve the diagnosis and treatment of the disease.

## 1. Introduction

Pyometra is a severe inflammatory condition affecting domestic cats, considered one of the most concerning reproductive diseases in felines [1,2]. This disorder is characterized by the accumulation of purulent exudate in the uterus [3], resulting from endometrial gland dilation, cystic structure formation, and accumulation of inflammatory cells, primarily neutrophils, macrophages, and lymphocytes [4,5]. In some cases, bacterial colonies associated with the inflammatory process can be identified [2,6]. Endometrial cells may exhibit signs of degeneration, and in chronic cases, fibrosis and proliferation of connective tissue in the uterine stroma may occur [7,8]. Pyometra presents a significant challenge for veterinarians and pet owners due to its insidious nature and potentially fatal outcomes, emphasizing the need for immediate medical intervention [1,2,7].

Pyometra is frequently diagnosed based on clinical signs observed by pet owners, including polydipsia, polyuria, lethargy, loss of appetite, vomiting, abnormal vaginal discharge, and abdominal distension [8,9]. These signs may be confused with other medical conditions, making accurate diagnosis crucial for effective treatment. Moreover, pyometra often requires emergency care, such as removal of the infected uterus and treatment of associated systemic infection [8]. Despite the severity of the condition, studies on surgical outcomes for queens treated with ovariohysterectomy (OHE) demonstrate highly favorable results [10]. Pailler et al. [10,11] reported a 100% survival rate to hospital discharge, with most queens recovering fully. Long-term outcomes are equally positive, with no significant reduction in life expectancy, even in older queens or those with comorbidities like mammary tumors or heart disease. However, severe cases involving uterine rupture may lead to peritonitis and sepsis, both life-threatening complications that can result in long-term sequelae such as infertility [10,11,12].

**Figure 1:**
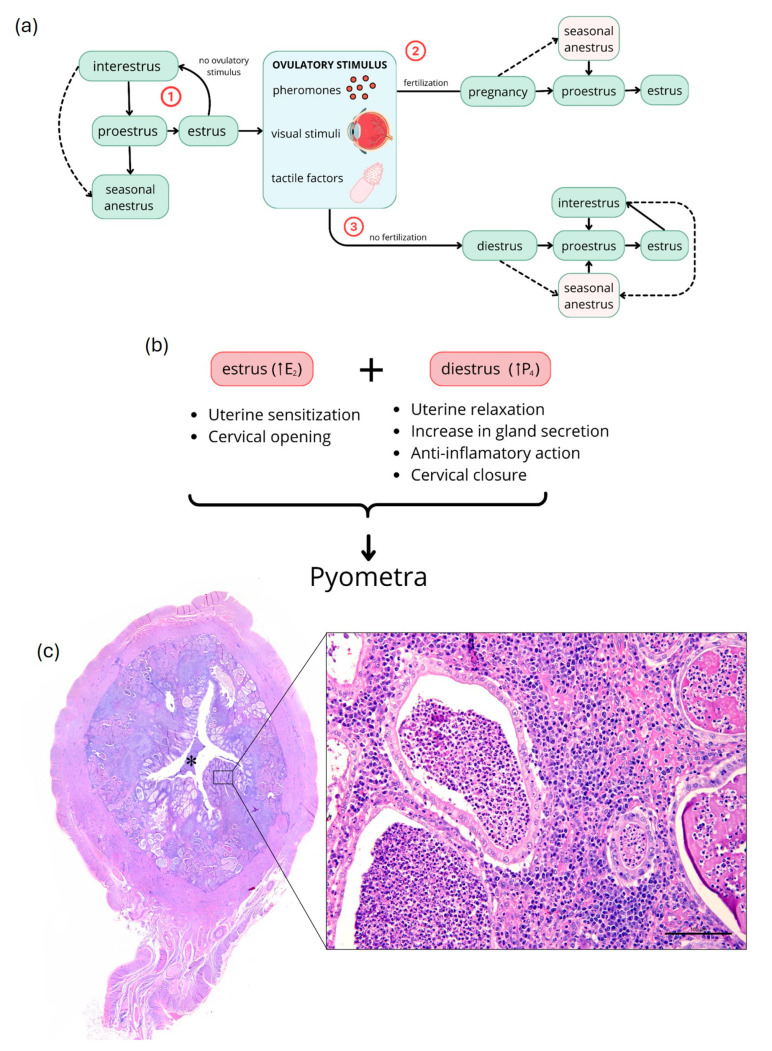
Schematic representation of the domestic cat estrous cycle and pyometra development. (**a**) Reproductive cycle of cats. The cycle begins with proestrus, followed by estrus, characterized by male receptivity and elevated E2 levels. Without ovulatory stimulus or spontaneous ovulation (1), cats return to proestrus, potentially experiencing brief interestrus or anestrus periods. In ovulatory cycles (2 and 3), fertilization may occur, resulting in pregnancy (2), lasting 63–65 days; without fertilization (3), cats enter diestrus, a luteal phase. Post-pregnancy and lactation, or after diestrus, cats return to proestrus through interestrus intervals or seasonal anestrus. (**b**) Hormonal factors favoring bacterial proliferation and pyometra development. During proestrus and estrus, increased E2 causes uterine sensitization and cervical opening, while in diestrus, increased P4 results in uterine relaxation, increased glandular secretion, anti-inflammatory action, and cervical closure. (**c**) Photomicrographs of uterus with grade 3 pyometra. Lower magnification: uterine cross-section showing glandular hyperplasia and ectasia (arrows) and interstitial inflammatory infiltrate (arrowhead) with pus accumulation in the uterine lumen (asterisk). Higher magnification (inset): glandular (asterisk) and interstitial (arrow) infiltration of neutrophils, lymphocytes, and macrophages. E2, estradiol; P4, progesterone; scale bar = 100 μm.

The disease primarily occurs in adult females during diestrus (Figure 1a), although it can also affect younger cats, often due to inappropriate administration of hormonal medications [2,5,13]. However, other factors, such as genetic predisposition and reproductive history, such as spontaneous ovulation, may play a significant role in its occurrence [7,14].

While data on occurrence, epidemiological profile, and clinical aspects of pyometra in cats are well-established, much remains to be elucidated regarding the molecular factors contributing to the disease’s development. Recent findings have revealed alterations in numerous factors involved in angiogenesis and inflammatory response within the uterus of cats with pyometra [15], potentially crucial for understanding the mechanisms underlying uterine infection. Given their potential as models for endangered wild felids [15,16], the study of reproductive diseases in domestic cats, such as pyometra, holds significant importance and warrants further investigation into associated mechanisms. This review aims to provide a comprehensive and up-to-date discussion of feline pyometra, exploring the molecular factors potentially involved in uterine and systemic alterations in affected animals.

## 2. Reproductive Cycle of Domestic Cats

The estrous cycle in female cats is divided into five distinct phases: proestrus, estrus, interestrus, diestrus, and anestrus (Figure 1a) [17,18]. However, variations in this presentation may occur due to their induced ovulation [17]. During proestrus, ovarian follicles begin to develop in response to follicle-stimulating hormone (FSH), resulting in a gradual increase in estradiol (E_2_) in preparation for estrus [18]. Estrus is the heat period characterized by receptive behaviors towards males and acceptance of coitus, with plasma E_2_ peaks exceeding 20 pg/mL [18]. Ovulation is generally induced by various stimuli, primarily tactile (Figure 1a), and occurs less frequently spontaneously [17,19]. If ovulation does not occur, cats may return to proestrus after a short interestrus period, or enter anestrus if under well-defined seasonal conditions (Figure 1a) [15,18]. If an ovulatory stimulus is followed by fertilization, cats become pregnant, with gestation lasting between 63 and 65 days [15,18] and plasma progesterone (P_4_) levels ranging from ~5.1 to 7.8 ng/mL between days 30 and 60 of pregnancy [20]. If fertilization does not occur after ovulation, cats enter diestrus (Figure 1a), a phase in which plasma P_4_ is also elevated due to corpus luteum formation [15,18]. During this period, cats are more susceptible to pyometra development due to elevated P_4_ levels [13]. After gestation (+lactation) or diestrus, cats typically return to proestrus following intervals of interestrus or seasonal anestrus (Figure 1a) [15,18].

## 3. Pyometra in Domestic Cats

Pyometra is one of the most prevalent reproductive diseases in female cats and can lead to sequelae for the animal, such as infertility and, in some cases, death [2,10,11,21,22]. It is characterized by the accumulation of inflammatory infiltrate, primarily neutrophils, lymphocytes, and macrophages (Figure 1c). Frequently, there may be fibrin exudation characterized by eosinophilic fibrillar material [5,6,22].

Based on histopathological characteristics, and the progression of the disease, pyometra in dogs and cats can be classified into four types, depending on the degree of pathological involvement and inflammatory infiltrate: type 1: endometrial hyperplasia without evidence of inflammatory process; type 2: endometrial hyperplasia accompanied by interstitial endometritis with mononuclear cells; type 3: exacerbation of endometrial inflammatory process, with neutrophil infiltration in the uterine lumen and endometrial glands (Figure 1c; asterisk); type 4: chronic endometritis with endometrial hypotrophy due to accumulation of inflammatory cells in the uterine lumen and, in some cases, squamous metaplasia of the epithelium [2,4].

In some cases of pyometra, ulceration of the endometrial epithelium may occur because of chronic inflammation and tissue damage. Additionally, epithelial and myometrial hypotrophy can develop when the accumulation of exudate in the uterine lumen increases pressure, compressing the epithelial cells and leading to their degeneration and reduction in size [14,23]. This process compromises the integrity and function of the endometrial epithelium, further exacerbating the inflammatory condition and contributing to more severe sequelae, such as reproductive dysfunction and infertility [2,12,24].

In types 3 and 4, cellular debris and mucinous material are commonly observed in the tissue. The superficial layers of the endometrium may occasionally exhibit reactions similar to those observed during pregnancy, such as decidual reaction and papilliform projections formation, a condition known as pseudoplacental hyperplasia [2,4]. Other findings include glandular hyperplasia and ectasia, characterized by an increase in the number and dilation of endometrial glands, as well as the presence of cysts in these glands, known as cystic endometrial hyperplasia (CEH) [4,25,26,27]. Furthermore, congestion and hyperemia in blood vessels are common observations. Lymphatic vessel ectasia may also be present. In more advanced cases, the inflammatory infiltrate can extend to the myometrium and perimetrium [2,7,9]. Figure 1c depicts a uterine lumen with superficial and deep endometrium, showing a marked inflammatory infiltrate predominantly lymphoplasmacytic, with intact and degenerated neutrophils, interspersed with eosinophilic fibrillar material, cellular debris, and weakly basophilic mucinous material. The infiltrate frequently extends to the epithelium and lumen of endometrial glands, which are sometimes ectatic and cystic, with hypertrophy of the lining epithelium.

Despite being a clinically significant reproductive disorder in cats, pyometra is reported more frequently in dogs [5,15,28]. This discrepancy likely stems from differences in luteal phase duration between the two species, variability in ovulation occurrence in domestic cats, as well as underdiagnosis and non-specific clinical signs in felines [5,27]. Generally, cats only ovulate following a sufficient luteinizing hormone (LH) surge triggered by vaginal stimulation during mating (Figure 1a) [15,26,28].

Various risk factors are associated with the occurrence of pyometra in cats. Age plays a significant role, with pyometra being more common in older, intact female cats over five years of age that experience regular and irregular hormonal cycles, although it can also affect younger cats. In the latter, it is typically associated with the exogenous administration of sex steroids as a contraceptive method [6,26,29]. Furthermore, the stage of the reproductive cycle is a determining factor, and pyometra is frequently diagnosed during diestrus when P_4_ levels are elevated [2,14,23]. Reproductive history is suggested to be a risk factor, as cats that have experienced multiple pregnancies, pseudopregnancies, or abortions may have a higher likelihood of developing pyometra [3,30]. In addition, the occurrence of spontaneous ovulation plays an important role in the pathogenesis of pyometra. While queens are considered induced ovulators, spontaneous ovulation has been reported, especially in certain circumstances of frequent sexual activity without copulation or hormonal imbalances [31,32]. This spontaneous ovulation can lead to prolonged luteal phases and elevated P_4_ levels, which create a favorable environment for bacterial proliferation in the uterus. P_4_ is known to reduce the immune response in the uterus, increase cervical closure, and promote endometrial glandular secretion, all of which contribute to the development of pyometra [8].

Breed predisposition in cases of pyometra in queens has been increasingly recognized, with certain purebred cats showing a higher risk of developing the condition [26,33]. Studies indicate that Sphynx cats are particularly susceptible, along with other breeds such as Siberian, Ocicat, Korat, Siamese, Ragdoll, Maine Coon, and Bengal. This suggests a possible genetic predisposition, supported by observations of familial clusters of the disease in geographically isolated populations [26]. However, data on the incidence of spontaneous ovulation within these breeds are still limited, and further studies are needed to explore its potential role in familial clustering and breed susceptibility.

Furthermore, the uterine microbiome plays an important role in the pathogenesis of pyometra, as reported in a study on bitches [34]. Alterations in microbial composition may facilitate bacterial colonization and proliferation in the uterus [35,36]. A recent study demonstrated that proteins in the uterine tissues of female dogs with pyometra exhibit different glycosylation patterns compared to healthy uterine tissues [34]. These findings suggest that proteins in the uterus of dogs with pyometra are glycosylated differently, which may impact the normal functions of uterine cells, potentially leading to a variety of pathological reactions, including altered cell communication and signaling [34,37]. Altered expression of glycoconjugates in the endometrial epithelium may therefore be a key factor in facilitating bacterial invasion, contributing to the development of pyometra [34,36]. However, studies in cats are needed to elucidate the role of the uterine microbiome in the development of pyometra.

## 4. Etiopathogenesis

The primary cause of pyometra in cats is related to uterine predisposition to bacterial colonization due to increased levels of E_2_ during the estrus phase and P_4_ during diestrus (Figure 1b) [2,14] Under cyclic conditions, increased E_2_ is essential for the proliferation of uterine glands. However, unusual elevation of E_2_ can cause cystic endometrial hyperplasia (CEH) and increase uterine sensitivity to P_4_ (Figure 1b) [38]. Additionally, stimulation of the cervical opening may allow ascending colonization by vaginal bacteria, making the uterus more susceptible to bacterial infection (Figure 1b) [2,7,14,15]. When P_4_ levels rise, uterine relaxation, decreased myometrial contractions, and cervical closure occur, creating a favorable environment for bacterial growth (Figure 1b) [2,5,14]. This combination of factors promotes the development of pyometra in dogs and cats [7,14,39].

Endocrine disturbances in the endometrium are recognized as risk factors, particularly for the development of CEH. This condition results in undesirable morphological and functional changes, primarily affecting the endometrial glands. Furthermore, excessive mucus secretion creates favorable conditions for abundant bacterial growth within the uterine lumen, as seen in bitches [38,40].

Bacteria play a crucial role in the etiopathogenesis of pyometra; uropathogenic *Escherichia coli* (*E. coli*) is the most frequently isolated Gram-negative bacterium from the uteri of cats and dogs with pyometra [41,42]. Furthermore, this microorganism is demonstrated to be the most isolated strain in the lower reproductive tract of healthy female cats. Other bacteria may also be involved in the development of pyometra, such as *Streptococcus* spp., *Staphylococcus* spp., *Pseudomonas* spp., *Proteus* spp., *Enterobacter* spp., *Nocardia* spp., *Pasteurella* spp., and *Klebsiella* spp. [2,5,14,41,42].

## 5. Molecular Aspects Related to the Occurrence of Pyometra in Domestic Cats

**Figure 2:**
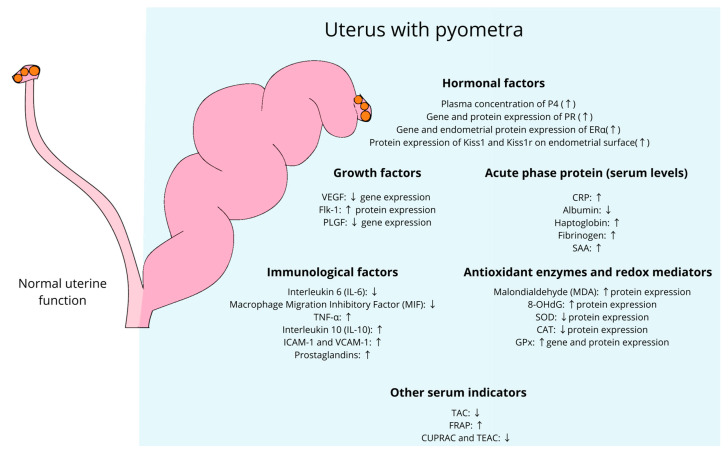
Plasma and uterine modulation of molecular factors involved in the occurrence of pyometra in domestic cats. P4, progesterone; PR, progesterone receptor; ERα, estrogen receptor α; Kiss1, kisspeptin; kiss1r, kisspeptin receptor; VEGF, vascular endothelial growth factor; flk-1, tyrosine kinase receptor 1; PLGF, placental growth factor; CRP, C-reactive protein; SAA, serum amyloid A protein; IL-6, interleukin 6; MIF, macrophage migration inhibitory factor; IL-10, interleukin 10; MDA, malondialdehyde; 8-OHdG, 8-hydroxy-2′-deoxyguanosine; SOD, superoxide dismutase; CAT, catalase; GPx, glutathione peroxidase; TAC, total antioxidant capacity; FRAP, ferric reducing ability of plasma; CUPRAC, cupric reducing antioxidant capacity; TEAC, trolox equivalent antioxidant capacity. Upward arrows (↑) indicate an increase in expression or activity of the respective factors, while downward arrows (↓) indicate a decrease.

Hormonal, immunological, redox, angiogenic, and growth mediators play crucial roles in the morphophysiology of the genital tract of domestic cats, contributing to various processes in the reproductive cycle and gestation [15]. Thus, alterations in the expression of these mediators may favor or be associated with the development of various reproductive pathologies, such as pyometra. These changes can not only result in subfertility or infertility, but also trigger the death of the affected individual. Understanding the complex interaction of these molecular factors is fundamental for the development of new effective diagnostic, preventive, and therapeutic approaches in the context of pyometra [14,15,23,42]. Figure 2 summarizes the main known molecular alterations in the uterus and plasma of cats with pyometra.

### 5.1. Hormonal Factors

Multiple hormonal factors modulate the estrous cycle, prepare for pregnancy, and maintain uterine homeostasis [15]. Understanding the modulation of these factors is crucial for elucidating the mechanisms involved in pyometra occurrence. Recent studies have described the expression profile of hormone receptors in this disease, including estrogen (ERα), progesterone (PR), androgen (AR) receptors, and other mediators, such as the kisspeptin/Kiss1r system [14,15,43].

#### 5.1.1. Sex Steroids and Receptors

##### Progesterone (P_4_)

P_4_ acts in the uterus through its receptors to control endometrial proliferation [13,44]. Alterations in these receptors have been associated with proliferative disorders such as endometrial hyperplasia in queens and bitches [15,29]. Recent studies have shown that cats with pyometra exhibit higher plasma P_4_ concentrations compared to healthy cats [14,45], suggesting that the uterine inflammatory response observed in pyometra may affect or result from abnormal corpus luteum function. While no studies have described the modulation of steroidogenic enzymes in the ovaries of cats with pyometra, an increase in 3β-hydroxysteroid dehydrogenase (3β-HSD), the enzyme responsible for progesterone synthesis from pregnenolone, has been observed in the uterus and corpus luteum of dogs with pyometra [46]. However, alterations in P_4_ receptor expression may also affect uterine signaling of this hormone. Indeed, in cats with pyometra, both protein and gene expression of PR are increased [14,47], similar to observations in dogs with pyometra [38,48].

##### Estrogen

Uterine estrogen receptors, primarily the alpha receptor (ERα), are key regulators of uterine function, including endometrial growth and endometrial gland proliferation, as demonstrated in knockout mice [49]. ERα protein expression is increased in the endometrium of cats with pyometra, as observed for the *ESR1* gene [13,47]. Despite this, no changes in plasma E_2_ profiles have been observed in these animals [14,45]. Moreover, although E_2_ elevation is critical for predisposing uterine conditions to CEH, this hormone does not appear to be important for the maintenance of pyometra. Thus, the upregulation of ERα in the uterus of cats with pyometra may be a result of the high production of pro-inflammatory cytokines, as there is a strong regulatory association between these factors [50]. 

##### Androgens

The androgen receptor (AR) is present in various cells of the female reproductive system, including uterine, ovarian, and placental cells. Under normal conditions, AR plays important roles in maintaining hormonal balance and regulating the reproductive cycle [51,52,53]. Although widely distributed throughout the luminal and glandular epithelium, no significant alterations in uterine AR gene and protein expression were identified in cats with pyometra [14].

#### 5.1.2. Kisspeptin

Kisspeptin is a peptide renowned for its role in regulating the hypothalamic–pituitary–gonadal (HPG) axis, directly influencing gonadotropin release [15] and, consequently, reproductive cycles. In domestic cats, beyond its description in the hypothalamus [54], studies have characterized its expression in the uterus [23,55], ovary [43,55], and placenta [56]. In mice, its importance in decidualization and endometrial adenogenesis has been demonstrated [57,58], including its influence on endometrial estrogenic signaling and the implantation process [59].

Studies evaluating kisspeptin and its receptor Kiss1r in cases of feline and canine pyometra are limited [23,60], although some have demonstrated the immunomodulatory role of kisspeptin in other reproductive diseases in mice [61,62]. The only study in cats with pyometra, conducted by Santos et al. [23], showed increased protein expression of Kiss1 and Kiss1r on the endometrial surface, while reduced expression was observed in deep glands. In dogs with pyometra, although no study has characterized the Kiss1/Kiss1r system in the uterus, Kurt et al. [60] described reduced plasma levels of kisspeptin in animals with cystic endometrial hyperplasia. Although it is not known whether these endometrial alterations in kisspeptin/Kiss1r system expression may be related to the cause or consequence of pyometra, there is strong evidence of their regulatory roles in angiogenesis and inflammation in the uterine alterations observed in women and mice, as reviewed by Zhang et al. [63].

#### 5.1.3. Oxytocin

Oxytocin, renowned for its uterotonic action, plays a crucial role in regulating uterine contractility and prostaglandin release [64]. The localization of oxytocin receptors (OTRs) across all uterine layers in canines, even under normal diestrus conditions, suggests its influence on sperm transport, secretion movement, and cervical dilation [29,64,65]. In cases of pyometra, despite reduced immunolabeling, the presence of OTRs indicates that the reproductive tract remains susceptible to this hormone’s action, even in pathological conditions. In felines, in addition to the administration of oxytocin stimulating the secretion of PGE by the feline endometrium at the beginning and middle of diestrus [66], oxytocin is reportedly used in combination with other drugs as a therapeutic approach for certain cases of dystocia, aiming to increase uterine tonicity [67]. However, to date, no study has evaluated the uterine modulation of oxytocin/OTRs in cats with pyometra.

### 5.2. Immunological Factors

The pathogenesis of pyometra involves a complex interplay of immunological, hormonal, and environmental factors. Previous studies using healthy diestrus animals as a reference have explored the underlying immunological mechanisms, highlighting involvement the crucial role of cytokines and other immune mediators in the development and progression of uterine infection in cats and dogs [56,68].

#### 5.2.1. Pro-Inflammatory Cytokines

Cytokines or interleukins are natural proteins or glycoproteins produced by the body that play central roles in activating or suppressing immune responses [69]. Pyometra, a uterine pathology associated with inflammatory processes, has been extensively studied in felines, characterizing the presence and regulation of cytokines. Regarding pro-inflammatory cytokines, Santos et al. [23] reported reduced uterine gene expression of interleukin 6 (*IL-6*) and macrophage migration inhibitory factor (*MIF*) in cats with pyometra, while tumor necrosis factor (*TNF*) α gene expression increased. Although controversial, the reduction of pro-inflammatory cytokines such as IL-6 in a situation of chronic inflammation may be related to several factors, such as receptor desensitization, negative regulation by anti-inflammatory cytokines, and even cellular exhaustion, since immune cells cannot maintain the same production rate indefinitely. Additionally, this study found no differences in endometrial protein expression of TNFα and MIF, or in interferon γ (INFγ) expression. Conversely, Abdelnaby et al. [70] observed increased endometrial immunostaining of TNFα in cats with pyometra, as well as nuclear factor kappa B (NF-κB) P65, a transcription factor regulating immune responses to infection [71]. Indeed, lipopolysaccharide (LPS) stimulation of feline uterine epithelial cell cultures increases TNFα secretion [72], while the combination of LPS + TNFα potentiates the synthesis of prostaglandins such as prostaglandin E2 (PGE_2_) and prostaglandin E2 alpha (PGE_2α_) [73], and elevates mRNA levels of *TNF* and *TNFR1* [74] in feline endometrial cell cultures.

#### 5.2.2. Toll-like Receptors

Toll-like receptors (TLRs) are essential components of the innate immune system, recognizing pathogen-associated molecular patterns (PAMPs) and initiating an immune response against bacterial, viral, and fungal pathogens [75,76]. In cats, nine TLRs (TLR1–9) have been identified, with TLR2 and TTLR4 being particularly relevant in recognizing bacterial components like lipoteichoic acid and lipopolysaccharides (LPSs) [76]. Pyometra has been shown to significantly influence TLR expression, especially in the endometrial epithelium. Studies have indicated that in queens with pyometra, there is increased expression of TLR2 and TLR4 in the uterine tissue, likely due to bacterial stimulation, such as *E. coli*, commonly isolated in such cases [2,77]. This heightened expression is associated with the inflammatory response, contributing to the influx of immune cells and cytokine production. Research has also demonstrated that hormonal treatments, such as medroxyprogesterone acetate (MPA), can alter TLR expression, potentially impairing the endometrium’s immune defenses, thereby increasing susceptibility to infections like pyometra [76]. These findings suggest that TLRs play a crucial role in the pathogenesis of pyometra, and their modulation by both bacterial infection and hormonal influences may be key factors in the disease’s development.

#### 5.2.3. Anti-Inflammatory Cytokines

Anti-inflammatory cytokines are signaling molecules of the immune system that help reduce inflammation and promote healing [69]. The main anti-inflammatory cytokines include interleukin-10 (IL-10) and transforming growth factor-β (TGF-β). The study by Santos et al. [23], in addition to characterizing the expression of various pro-inflammatory factors in the uterus of cats with pyometra, demonstrated increased endometrial immunostaining of IL-10, but without alteration in *IL-10* gene levels. The increase in IL-10 in the context of pyometra in cats may suggest a tissue response to the increase in pro-inflammatory cytokines, aiming to reestablish the local immune microenvironment and prevent damage caused by an exacerbated inflammatory environment.

#### 5.2.4. Prostaglandins

Prostaglandins (PGs) are lipid mediators that act as paracrine chemical messengers in processes such as inflammation, vasodilation/vasoconstriction, coagulation, and reproduction, including parturition [78,79]. The main types of PGs produced are prostaglandin I2 (PGI_2_), PGE_2_, and prostaglandin F2α (PGF_2α_) [79]. In cats with pyometra, increased plasma concentrations or tissue culture levels of PGE_2_ and PGF2α [73] and their metabolite 15-keto-(13,14)-dihydro-PGF2α (PGFM) [80], as well as PGI_2_ [77], have been observed. Additionally, upregulation of gene expression for PGF_2α_-synthase (*PGFS*) and prostaglandin-endoperoxide synthase 2 (*PTGS2*) has been reported in endometrial tissue [77].

The increase in various PG types, both in circulation and uterine tissue of cats with pyometra, may be crucial for the expulsion of purulent uterine content due to the role of these factors in uterine contraction [30,81], particularly in cases of open-cervix pyometra, as well as in the lysis of the corpus luteum itself, a gland necessary for maintaining plasma P_4_ levels. Indeed, this may even indicate their potential use in therapeutic approaches, as previously described [30,82]. Although the increase in PGs is primarily triggered via cyclooxygenase (COX) enzymes, as observed in bitches [83,84], few studies have evaluated this pathway in cats with pyometra. Saraiva et al. [85] reported a marginally significant difference (*p* = 0.05) in cyclooxygenase-2 (COX-2) expression in cats with cystic endometrial hyperplasia, while Jursza et al. [72] found that the increase in PGs caused by TNFα in cultured endometrial tissue from cats was suppressed by nimesulide (NS), a COX-2 inhibitor.

### 5.3. Growth Factors

Growth factors are molecules that modulate cellular proliferation, differentiation, and protein synthesis in the uterus throughout the reproductive cycle. These include epidermal growth factors (EGFs), insulin-like growth factors (IGFs), fibroblast growth factors (FGFs), transforming growth factors (TGFs), and vascular endothelial growth factor (VEGF) [15,71,86,87]. During uterine inflammatory processes, such as pyometra, these factors may be associated with the regulation of immune responses, angiogenesis, and tissue repair [21]. However, few growth factors have been studied in feline pyometra [23].

#### 5.3.1. Vascular Endothelial Growth Factor (VEGF)

VEGF is well-established for its role in uterine angiogenesis, as demonstrated in mice [88]. A study by Santos et al. [23] observed reduced endometrial expression of the *VEGF* gene in cats with pyometra, although no change was noted in immunostaining. Conversely, the same study demonstrated increased endometrial protein expression of tyrosine kinase receptor 1 (Flk-1/KDR/VEGFR2) [23], the primary receptor for VEGF [88]. Additionally, a reduction in gene expression of placental growth factor (*PLGF*) was observed in cats in this study [23], which, unlike VEGF, is a factor more commonly associated with vascular maturation, as reviewed in experimental models [89]. Collectively, these findings suggest that pyometra in cats could be associated with dysregulation of mediators involved in blood vessel formation and maturation in the endometrium. Chronic inflammation may lead to downregulation of VEGF, as observed in the study by Santos et al. [23], while increased Flk-1 staining may be a compensatory response to attempt to maintain angiogenesis and vascular integrity [26]. However, studies confirming these hypotheses are needed.

#### 5.3.2. Transforming Growth Factor (TGF)

Transforming growth factors (TGFs) are a group of cytokines that regulate various biological processes [90]. In the uterus, they modulate endometrial development and inflammation throughout the menstrual/estrous cycle, embryo implantation, and maintain tissue homeostasis, as shown in bitches [88,89,91,92], thus preserving uterine integrity. While studies have demonstrated increased expression of TGF-β1 and TGF-β3 and decreased TGF-β2 in canine pyometra compared to control groups, no investigations have been conducted on these mediators in feline pyometra [86,93].

#### 5.3.3. Epidermal Growth Factor (EGF)

The epidermal growth factor (EGF) is essential for cell proliferation, migration, and differentiation [92]. In the uterus, it plays a crucial role in epithelial maturation, endometrial development, and wound healing. Uterine EGF expression fluctuates throughout the estrous cycle and is influenced by E_2_ and P_4_ hormonal levels, with increased endometrial immunostaining observed in cats following treatment with these hormones [86,87,91]. However, studies investigating this factor in cats with pyometra are currently lacking. In contrast, increased gene and protein expression of the epidermal growth factor receptor (EGF-R) have been demonstrated in dogs with pyometra [87].

### 5.4. Redox Mediators

Oxidative stress, characterized by an imbalance between reactive oxygen species (ROS) production and antioxidant defenses [94], has been associated with uterine alterations observed in canine pyometra [7]. Antioxidant factors play a crucial role in neutralizing ROS, protecting cells and tissues against oxidative stress-induced damage. Enzymes such as superoxide dismutase (SOD), catalase (CAT), and glutathione peroxidase (GPx), along with antioxidant molecules such as glutathione, are vital in maintaining redox balance [95,96]. In pyometra cases, evidence suggests an increase in redox mediators, indicating the presence of oxidative stress in the uterus and adjacent tissues. Furthermore, antioxidant capacity may be compromised due to ROS overload resulting from the inflammatory response and bacterial accumulation in the uterus. This can lead to a vicious cycle where oxidative stress perpetuates inflammation and vice versa [7,14,94].

#### 5.4.1. Pro-Oxidant Factors

Malondialdehyde (MDA), a byproduct of lipid peroxidation, results from free radical attacks on polyunsaturated fatty acids in cellular membranes [97,98]. Commonly measured as thiobarbituric acid reactive substances (TBARS), MDA serves as a biomarker for oxidative stress and consequent damage to cellular membrane integrity [98]. In feline pyometra cases, Abdelnaby et al. [70] and Abdelbaset et al. [45] demonstrated elevated serum MDA levels, correlated with decreased total antioxidant capacity (TAC) [45,70]. Furthermore, Nascimento et al. [14] observed increased endometrial and myometrial immunostaining of 8-Hydroxy-2′-deoxyguanosine (8-OHdG), a biomarker of oxidative DNA damage. Collectively, these studies confirm a state of uterine oxidative stress in domestic feline pyometra cases.

#### 5.4.2. Antioxidant Factors

Among the most evaluated antioxidant factors in studies involving oxidative stress are the antioxidant enzymes SOD, CAT, and GPXs [99]. In this context, Abdelbaset et al. [45] demonstrated a reduction in plasma SOD concentration in female cats with pyometra, while Nascimento et al. [14] observed no differences in uterine protein and gene expression in cats with this condition. SOD acts in the neutralization of free radicals, especially the superoxide anion (O_2_^•−^) [13,100]. Under normal conditions, SOD converts superoxide into hydrogen peroxide (H_2_O_2_), which is subsequently decomposed by other antioxidant enzymes, such as CAT and GPx [100,101]. This reduction in serum SOD concentration observed by Abdelbaset et al. [45] may contribute to the increased oxidative stress associated with pyometra in cats and dogs [7,14,102].

Although no differences were observed in uterine SOD expression, Nascimento et al. [14] reported reduced endometrial CAT immunostaining in cats with pyometra, while Abdelbaset et al. [45] demonstrated serum CAT values twice lower in cats with pyometra compared to healthy cats, confirming the reduction of this enzyme in pyometra [13,45]. Catalase acts by decomposing H_2_O_2_, a potentially harmful byproduct of cellular metabolism, into water and oxygen [99], thus preventing the accumulation of H_2_O_2_, which is also harmful to cells [7,95].

Regarding GPx, similar to CAT, it is an antioxidant enzyme that plays a crucial role in neutralizing peroxides, including H_2_O_2_, in the organism [99,103]. Unlike what was observed in dogs [102], cats with pyometra exhibit increased protein and gene expression of GPX1 in the uterus [104], as well as increased plasma GPX levels [45]. The elevation of GPX1 in the uterus of cats with pyometra may represent a tissue response to oxidative stress aimed at restoring redox homeostasis [104].

In addition to antioxidant enzymes, a previous study evaluating total antioxidant capacity (TAC) has demonstrated reduced serum concentrations of ferric reducing ability of plasma (FRAP) in cats with pyometra, as well as decreased levels of cupric reducing antioxidant capacity (CUPRAC) and trolox equivalent antioxidant capacity (TEAC) [94]. Furthermore, significant correlations between these serum antioxidants (Thiol, CUPRAC, TEAC2) and acute phase proteins, such as haptoglobin (Hp) and serum amyloid A (SAA), have been observed in cats with pyometra, suggesting that acute phase proteins (APPs) may serve as prognostic biomarkers for pyometra in this species [94,105].

### 5.5. Acute Phase Proteins (APPs)

Acute phase proteins (APPs) are a class of proteins that play a crucial role in an organism’s response to inflammatory conditions, such as pyometra [94,106]. Their blood concentration fluctuates in response to inflammatory processes, serving as sensitive indicators of disturbances in the immune system [105].

Among serum proteins and inflammatory markers, C-reactive protein (CRP) is the first APP to increase at the onset of any infection or inflammation [107], and is frequently used as a sensitive marker of inflammatory processes. In female cats with pyometra, Abdelbaset et al. [45] described an increase in CRP, suggesting its use as a serum biomarker for this disease.

Albumin, unlike CRP, is considered a negative APP, as its circulating concentrations may decrease during an inflammatory process [108]. Indeed, cats with pyometra or other inflammatory conditions exhibit reduced plasma albumin levels [70,80,94,109]. Since this protein primarily functions in regulating osmotic pressure, substance transport, and pH control, a reduction in its quantity can result in edema, which is characteristic of an inflammatory process [108,110].

Haptoglobin (Hp) is an acute phase protein that can increase in response to inflammation and infection. Studies have demonstrated elevated haptoglobin levels in cats with pyometra [94] and in other disease models [111]. This protein plays a crucial role by binding to hemoglobin, forming a haptoglobin–hemoglobin complex, which prevents iron uptake by microbes, thereby inhibiting their replication and survival. Furthermore, Hp can bind to free hemoglobin, preventing its oxidation with lipids and proteins, which explains the reduction of Hp in cases of hemolysis [112].

Fibrinogen is a crucial protein in blood coagulation [109], and its levels may increase as part of the organism’s response to inflammation in cases of inflammation or infection [5,105,109]; however, studies in cats with pyometra have not yet been conducted.

Serum amyloid A (SAA) is another APP that can increase in response to various systemic inflammatory conditions. Elevated SAA levels have been observed in cats with pyometra [94,109,111], as previously demonstrated in bitches and mares with the same disease [113,114]. Due to its functions in the immune response, an increase in this factor may be crucial for recruiting immune cells to sites of inflammation and inducing the activity of enzymes that degrade the extracellular matrix [114].

## 6. Conclusions

Pyometra in cats is a severe reproductive disease that still requires a better understanding of its epidemiology, etiopathogenesis, and progression. While numerous studies have been conducted in dogs, there remains a significant knowledge gap regarding this condition specifically in felines. Furthermore, research evaluating the molecular aspects involved in the development and pathogenesis of this disease in cats is necessary, as it will enable the development of more effective prevention, diagnostic, and treatment strategies.

## Data Availability

No new data were created or analyzed in this study. Data sharing is not applicable to this article.
